# Influence of awakening from general anesthesia on the distribution of glucose 2.5% with balanced electrolytes in adults

**DOI:** 10.3389/fmed.2025.1577418

**Published:** 2025-06-04

**Authors:** Robert G. Hahn, Fredrik Sjöstrand

**Affiliations:** ^1^Karolinska Institutet at Danderyds Hospital (KIDS), Stockholm, Sweden; ^2^Department of Clinical Research and Education, Karolinska Institutet at Södersjukhuset, Stockholm, Sweden

**Keywords:** hemodilution, fluid balance, infusion, pharmacokinetics, glucose solution, laparoscopic cholecystectomy, plasma glucose

## Abstract

**Background:**

The glucose-free crystalloid fluid used for intravenous infusion during surgery might be combined with or replaced by 2.5% glucose in debilitated patients. The aim of the present study was to use kinetic modeling to quantify the distribution of 2.5% glucose with balanced electrolytes administered during and after general anesthesia. The hypothesis was that awakening from anesthesia changes glucose and/or fluid volume kinetics in distinct ways.

**Methods:**

A secondary analysis was performed based on data derived during and after intravenous administration of an isotonic mixture of 20 mL/kg of glucose 2.5% with electrolytes over 60 min in 12 non-diabetic adult patients undergoing laparoscopic cholecystectomy. Population glucose and volume kinetic analyses were performed based on blood and urine data collected for 4 h, which included 2 postoperative hours. Periods before and after awakening from anesthesia were contrasted using covariate analysis and further compared to 35 infusions in 17 awake volunteers who had received the same fluid at different rates and volumes.

**Results:**

The return flow of distributed fluid to the plasma was strongly retarded during and after anesthesia and promoted peripheral edema. Awakening decreased the rate of distribution, which counteracted the additional build-up of this edema but expanded the plasma volume. Urine output was strongly dependent on the mean arterial pressure; the urine flow rate at 75 mmHg was only 22% of the flow rate obtained at 90 mmHg. Simulations showed that the rate of administration of glucose 2.5% should be < 500 mL over 30 min to maintain plasma glucose < 10 mmol/L during surgery and postoperatively.

**Conclusion:**

Awakening from general anesthesia inhibits the distribution of 2.5% glucose solution and accelerates the return of distributed fluid, with both responses increasing the plasma volume. These two effects counteract postoperative development of hypovolemia.

## 1 Introduction

Crystalloid solutions with nearly isotonic electrolyte contents are the most widely used infusion fluids during general anesthesia and surgery. One alternative fluid is 2.5% glucose containing a balanced concentration of electrolytes (2.5% Glu+). This fluid might be even more suitable in physically weak patients with an unclear capacity for glycogenolysis and in patients with diabetes. Indeed, some hospitals provide 2.5% Glu+ to all patients to hydrate the intracellular space because the daily loss of volume of approximately 700 mL due to evaporation (1) can be exacerbated by additional volume losses due to diuretic therapy, sweating, sodium overload, diarrhea, and hyperpyrexia. The benefits of administering exogenous glucose include the prevention of hypoglycemia, blunting of starvation, and potential improvements in postoperative well-being. The downside is the potential development of hyperglycemia if too much glucose is infused too rapidly (2–4). Therefore, anesthetists should provide glucose cautiously and, at best, in addition to the infusion of other fluids.

Another point to consider is that glucose metabolism (5) and fluid kinetics (6) differ between the conscious state and during surgery performed under general anesthesia. Furthermore, because the residual effects of the anesthesia may remain in the early postoperative period, the kinetics cannot be assumed to be the same as in the fully awake or anesthetized states. Therefore, the aim of the present study was to describe how the change from the anesthetized to the conscious state alters the turnover of a glucose-containing fluid. The study also included awake volunteers who had not undergone surgery to highlight whether the kinetics of 2.5% Glu+ in the postoperative period falls between the kinetics observed in the anesthetized and awake states. For clarity, we use the terms awake, anesthetized, and postoperative from this point onward to indicate healthy individuals, patients during surgery, and the same patients after surgery, respectively.

The *primary hypothesis* was that the volume and glucose kinetics change as a patient awakens from general anesthesia (anesthetized vs. postoperative states). The *secondary hypothesis* was that the volume and glucose kinetics of 2.5% Glu+ differ between the awake and anesthetized states. We also used kinetic data from a previous “twin” study of Ringer’s solution to examine whether the combination of these two fluids can stabilize the plasma volume expansion during awakening (7).

## 2 Materials and methods

### 2.1 Subjects

This report is a secondary analysis of three prospective studies in which subjects received 2.5% Glu+ . All data were collected in the same way.

The first study included administration of 2.5% Glu+ to 12 patients undergoing elective laparoscopic cholecystectomy (8). No other infusion fluid was given. The duration of the study was 4 h, with the anesthesia ending after approximately 2 h. The 2 h postoperative period, which is the key focus of the present study, has not been analyzed previously.

The second and third series included 35 infusions of 2.5% Glu+ in 17 healthy volunteers (9, 10). In the first group, each volunteer received only one infusion. The purpose of the second series was to challenge the model linearity by providing different volumes at various infusion rates on 4 occasions to 6 volunteers, for a total of up to 24 experiments. This analysis confirmed that the kinetic parameters were valid for a wide range of infusion regimens.

The protocols for the original studies were approved by the Ethics Committee of Huddinge University Hospital (Dnr. 428/97, 429/97, and 258/00). Each subject provided informed consent to participate before any infusion was initiated. None of the subjects had daily medication or a family history of diabetes.

### 2.2 Procedure

Surgical patients. After an overnight fast, the infusion experiments were initiated in the morning at approximately 8:30 a.m. The patients received premedication with 2.5–7.5 mg of ketobemidone by intramuscular injection 1 h before anesthesia. A cannula was placed in the cubital vein of each arm—one cannula was used for fluid infusion and the other for blood sampling. Urine was collected and quantified via a catheter placed in the bladder.

General anesthesia included tracheal intubation and was maintained with continuous administration of sevoflurane and intravenous (IV) injections of fentanyl and rocuronium. Pneumoperitoneum was maintained with the patient in the reverse Trendelenburg position.

Upon initiation of surgery, an IV infusion of 20 mL/kg of 2.5% Glu+ (sodium 70, chloride 45, and acetate 25 mmol/L; Rehydrex, Pharmacia, Uppsala, Sweden) was given at a constant rate over 60 min via an infusion pump.

Volunteers. The volunteers were also given 2.5% Glu+, but the amount varied between 10, 15, and 25 mL/kg and the infusion time was 30 or 60 min. They voided just before the infusion started and, while in the supine position, whenever they experienced urgency during the experiments.

### 2.3 Measurements

Venous blood samples were collected every 5 min for 90 min and thereafter every 10 min for measurement of the blood hemoglobin (Hb) concentration, red cell count, and mean corpuscular volume using a Technicon Advia 120 (Bayer, Tarrytown, NY, United States), with coefficients of variation of 1.0, 1.2, and 0.5%, respectively.

Plasma dilution was calculated based on the mean value of the dilution of the Hb and red cell count, which was corrected for any change in the size of the red cells (8). Finally, the obtained expression was divided by (1–baseline hematocrit) to convert the dilution of whole blood (i.e., the hemodilution) to a plasma dilution.

Plasma glucose was sampled on the same occasions and measured using a Hitachi 917 analyzer (Hitachi Co., Naka, Japan).

Non-invasive arterial pressure was monitored in the arm not used for fluid infusion using an automatic device (Datex-Ohmeda, Box 900, Finland).

### 2.4 Kinetic analysis

**FIGURE 1:**
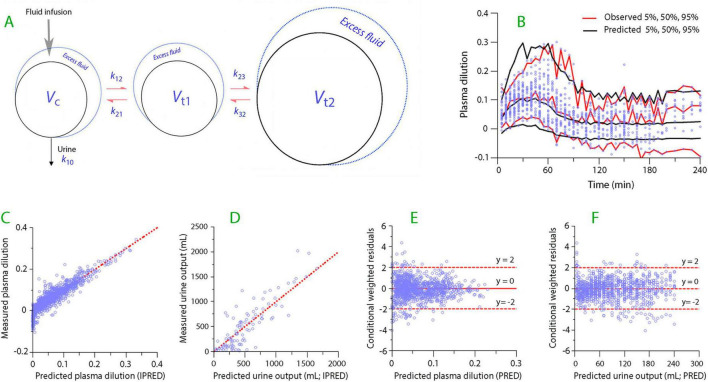
Kinetic model and goodness-of-fit for analysis of the infused fluid. **(A)** Schematic drawing of the kinetic model used in the study. **(B)** Predictive check. The measured data points are shown together with the observed and predicted confidence intervals. **(C)** The relationship between the measured plasma dilution and the model-predicted plasma dilution when the covariates are considered. (**D**) Same plot but for the model-predicted urine output. **(E)** The conditional weighted residuals (CWRES) for the plasma dilution when predicted without consideration of the covariates. **(F)** Same plot but for the urine output.

Volume kinetics was studied by fitting a three-volume “base model” with five rate constants (*k*_12_, *k*_21_, *k*_23_, *k*_32_, and *k*_10_) and one scaling factor between dilution and volume (*V*_*c*_, central volume) to the urinary output and the plasma dilution. These two measured variables served as input data for the calculations. A graphical illustration of the model is shown in Figure 1A.

Briefly, fluid is infused into the plasma (*V*_*c*_). Distribution occurs to a fast-exchange interstitial space (*V*_*t*1_) from which it either returns to *V*_*c*_ or becomes further distributed to a more remote interstitial, slow-exchange fluid space (*V*_*t*2_, the “third fluid space”) (11). The elimination rate constant (*k*_10_) was calculated as the collected urine volume divided by the area under the curve of the volume expansion of *V*_*c*_ over the period during which the plasma was sampled.

The estimates of the six model parameters (i.e., the 5 rate constants and *V*_*c*_) could be modified by individual-specific *covariates*, such as body weight or arterial pressure. Covariates were also used to quantify the extent to which the typical parameter values differed in the anesthetized and postoperative states compared to the awake state (12). Appropriate covariates were identified by reviewing “eta plots” for all combinations of parameters and covariates. The following covariates were examined: Body weight, mean arterial pressure, plasma glucose, anesthetized period, and postoperative period.

The choice of kinetic model, the differential equations used to describe the chosen kinetic model, and the mathematical determination of the covariate effects on the model parameters are explained in Supplementary File 1.

### 2.5 Simulations

The plasma glucose concentration and distribution of the infused fluid volume between *V*_*c*_, *V*_*t*1_, and *V*_*t*2_ in the three settings were simulated by inserting the optimal parameter values derived by the analyses into the Phoenix program. Simulations were also performed using the optimal parameter values from a previous study of the intra- and postoperative phases in which Ringer’s acetate had been administered as the only infusion fluid (7). Key data from that study are given in Supplementary File 1.

### 2.6 Endpoints

The *primary endpoint* was a description and quantification of the differences in the volume and glucose kinetics of 2.5% Glu+ between the anesthetized and postoperative periods. The *secondary endpoint* was a comparison of the volume and glucose kinetics between the anesthetized and awake states.

### 2.7 Statistics

The selection of the kinetic model was guided using the Akaike criterion. All measurements of plasma dilution and urinary excretion were simultaneously fitted to the kinetic model (including the base model and the covariates) using Phoenix software version 8.3.4 for non-linear mixed effects (Phoenix NLME, Pharsight, St. Louis, MO) with First-Order Conditional Estimation Extended Least Squares (FOCE ELS) as a search routine. This routine operates slowly but provides very precise parameter estimates.

The kinetics of the administered glucose molecules was studied separately using the one-compartment model implemented in the Phoenix program (Supplementary File 1).

Kinetic parameters were reported as the best estimate and 95% confidence interval (CI) according to the output from the Phoenix program. The significance levels for inclusion of the covariates were taken from Phoenix. In general, a covariate is significant at *P* < 0.05 if its inclusion decreases the −2 log likelihood (−2 LL) for the model by > 3.84 points and at *P* < 0.01 if −2 LL decreases by *P* < 0.01 (12)

The typical value (tv) reported in the tables represents the parameters valid for the awake state. Statistically significant covariates served as mathematical tools for distinguishing the kinetics of the anesthetized and postoperative states from the kinetics of the awake state.

The behavior of the models (goodness-of-fit) was studied using residual plots, conditional weighted residuals (13), and predictive checks.

Demographic data showing a normal distribution were reported as the mean and standard deviation (SD), and differences were evaluated by one-way analysis of variance (ANOVA). Significance is reported if *P* < 0.05

## 3 Results

### 3.1 Subjects and experiments

The demographic and baseline data are shown in Table 1. The arterial pressures and Hb concentrations were significantly lower when the infusion was initiated in the anesthetized state, which reflects the physiological adaptation to general anesthesia (14, 15).

**TABLE 1:** Demographic and baseline data.

Variable	Awake volunteers (*N* = 17)	During anesthesia (*N* = 12)	*P*-value
Infusions (*N*)	35	12	
Age (years)	30 ± 4	40 ± 8	0.001
Body weight (kg)	77 ± 14	75 ± 11	0.74
Blood hemoglobin (g/L)	138 ± 7	124 ± 16	0.001
Plasma glucose (mmol/L)	5.1 ± 0.3	5.1 ± 0.7	0.70
Plasma insulin (μmol/L)	37 (29–57)	35 (22–36)	0.17
Systolic arterial pressure (mmHg)	121 ± 10	105 ± 15	0.001
Diastolic arterial pressure (mmHg)	73 ± 8	61 ± 14	0.001
Mean arterial pressure (mmHg)	89 ± 7	76 ± 13	0.001
Heart rate (bpm)	63 ± 10	72 ± 8	0.001
Infused volume (mL)	1,246 ± 406	1,423 ± 200	0.16
Infusion rate (mL/min)	29 ± 11	24 ± 3	0.08

Data are the mean ± SD and one-way ANOVA used for statistics except for insulin, which is reported the median (25th–75th percentile range) and evaluated by Mann-Whitney’s U test.

The 47 studied experiments comprised 1,088 data points of dilution/glucose and 95 urine collections. The mean operating time was 117 ± 40 min, and the mean postoperative follow-up continued for 123 ± 41 min. The volunteer experiments lasted for a mean time of 174 ± 20 min.

### 3.2 Kinetic analyses

The three-volume model was fitted to the data on plasma dilution and urine output from all subjects (Figure 1A). The final parameter estimates are given in Table 2, and performance measures are shown in Figures 1B–F.

**TABLE 2:** Population kinetic parameters for infused fluid volume in the final model.

	Covariate	Covariate model	Best estimate	95% CI	CV%	−2 LL
tv*V*_*c*_ (L)			3.12	2.68–3.55	7.1	
tv*k*_12_ (10^–3^ min^–1^)			41.3	33.9–48.7	9.1	
tv*k*_21_ (10^–3^ min^–1^)			12.8	10.4–15.3	9.7	
tv*k*_23_ (10^–3^ min^–1^)			1.78	1.54–2.01	6.7	
tv*k*_32_ (10^–3^ min^–1^)			3.82	3.66–3.98	2.1	
tv*k*_10_ (10^–3^ min^–1^)			16.2	13.5–18.9	8.6	−2457
*k*_10_	MAP	Power	8.44	7.40–9.49	6.3	−2683
*k*_12_	Plasma glucose	Power	−0.25	−0.29 to −0.22	−7.0	−2712
*k*_21_	Anesthesia	Exponential	−9.02	−11.7 to −6.34	−15.1	−2734
*k*_21_	Postoperative	Exponential	−1.92	−2.57 to −1.27	−17.3	−
*k*_12_	Anesthesia	Exponential	0.25	0.06–0.44	38.5	−2771
*k*_12_	Postoperative	Exponential	−3.06	−4.06 to −2.05	−16.7	−

Both awake, anesthetized and postoperative patients are included. Shown are the typical values (tv) for the fixed parameters in the awake subjects, followed by individual-specific covariates that specify the factors that modify the typical values. The full equations for the parameters affected by covariates are to indicate that the data follow right here. *k*_12_ = 0.0413 (plasma glucose/7.86) ^–0.25^ (e ^awake=0,^ ^anesthesia = 0.25,^ ^postoperative = –3.06^) *k*_21_ = 0.0128 * (e ^awake=0, anesthesia = –9.02, postoperative = –1.92^) *k*_10_ = 0.0162 * (MAP/86.6)^8.44^ tv, typical value for the group; CI, confidence interval; CV%, coefficient of variation (inter-individual); LL, log likelihood for the model during development. Decrease by > 6.6 points = *P* < 0.01. Mean MAP (mean arterial pressure) 86.6 mmHg, Mean plasma glucose 7.86 mmol/L.

The central volume (the plasma) averaged 3.12 L.

Covariate analysis showed that urine output increased with the mean arterial pressure.

The return flow of the distributed fluid to the plasma (*k*_21_) was inhibited during anesthesia, and some of this inhibition remained postoperatively.

The rate of distribution of fluid from the plasma to the interstitial space (*k*_12_) was strongly retarded during the postoperative period, and this retardation promoted hypervolemia.

**FIGURE 2:**
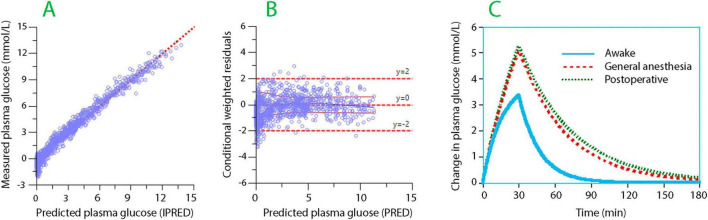
Goodness-of-fit and simulation for the analysis of plasma glucose. **(A)** The relationship between the measured plasma glucose and the model-predicted plasma glucose when the covariates are considered. **(B)** The conditional weighted residuals (CWRES) for the plasma dilution when predicted without consideration of the covariates. **(C)** Simulations of an intravenous infusion of 500 mL of 2.5% Glu+ over 30 min in three different settings (awake, anesthetized, and postoperative). The y-axis shows the increase in plasma glucose from the baseline, which averaged 5.1 mmol/L. Based on the kinetic data shown in Table 3.

The final parameter estimates for the analysis of glucose are shown in Table 3, and the associated performance measures are shown in Figures 2A,B. Here, the rate of elimination of glucose was lower during the surgery (−57%) and during the postoperative period (−62%) than in the fully awake state.

**TABLE 3:** Population kinetic parameters for infused glucose in the final model. The data on volunteers and anesthetized patients were pooled and analyzed simultaneously.

Kinetic parameter	Covariate	Covariate model	Best estimate	95% CI	CV%	−2 LL
tv*V*_*c*_ (L)			9.57	9.01–10.1	3.0	
tv*k*_10_ (10^–3^ min^–1^)			59.8	49.8–69.8	8.6	5,242
*k*_10_	Anesthesia	Exponential	−0.85	−1.20 to −0.51	−20.7	2,419
*k*_10_	Postoperative	Exponential	−0.97	−1.59 to −0.35	−32.6	− “−
*V*_*c*_	Body weight	Power	0.60	0.26–0.94	29.2	2,407

The Akaike criterion was 5,252 (one-compartment model) and 4,270 (two-compartment). The one-compartment model was still chosen because *V*_*c*_ was only 1.6 L and could not adequately reflect plasma glucose. tv, typical value for the group; CI, confidence interval; CV%, coefficient of variation (inter-individual); LL, log likelihood for the model during development. Decrease by > 6.6 points = *P* < 0.01. Mean body weight 77 kg.

### 3.3 Simulated infusion, single setting

Simulations of infusions given in a single setting only (awake, anesthetized, or postoperative), based on the kinetic data shown in Table 3, showed that 500 mL of 2.5% Glu+ could be administered over 30 min both during anesthesia and postoperatively without exceeding a plasma glucose level of 10 mmol/L (Figure 2C).

**FIGURE 3:**
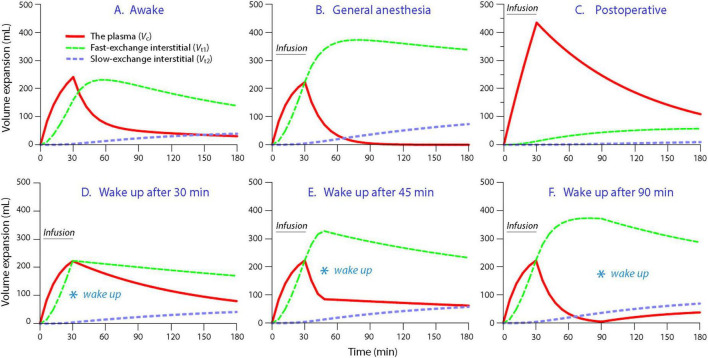
Simulations of fluid distribution. **(A)** Infusion of 500 mL of 2.5% Glu+ over 30 min in the awake state. **(B)** Same infusion given during general anesthesia. **(C)** Same infusion given during the postoperative phase. (**D**) The patient wakes up from anesthesia at the same time as the infusion ends (at 30 min). **(E)** The patient wakes up from anesthesia 15 min after the 30 min infusion ends. **(F)** The patient wakes up from anesthesia 60 min after the 30 min infusion ends.

Simulations based on the volume kinetic data from Table 2 showed that general anesthesia induced a greater volume expansion of *V*_*t*1_ than was observed in the awake state (Figures 3A,B). Furthermore, the low *k*_12_ after surgery resulted in a much greater expansion of *V*_*c*_ than was observed in the awake volunteers (Figure 3C).

The predicted urine output at 3 h was 292, 88, and 324 mL in the awake, anesthetized, or postoperative settings, respectively. The urine output varied with the mean arterial pressure, but the pressure showed limited variation between the three settings (means being 89, 83, and 79 mmHg, respectively).

### 3.4 Anesthesia and postoperatively

Simulations were also performed after integration of the kinetics during and after surgery, as is the case in real life. The duration of the plasma volume expansion became prolonged if the patient woke up from anesthesia at the same time as the 30 min infusion of 2.5% Glu+ ended (Figure 3D), whereas any period without infusion before awakening from the anesthesia promoted hypovolemia (Figures 3E,F).

By contrast, an infusion during surgery that continued into the postoperative period created an abrupt increase in the plasma volume expansion on awakening. A calculation shows that an increase from 135 to 460 mL would occur if 1 L of 2.5% Glu+ was infused over 2 h and awakening occurred at 1 h.

### 3.5 Combining 2.5% Glu+ with Ringer’s

We also explored combinations of 2.5% Glu+ and Ringer’s using kinetic data from a similar publication where Ringer’s acetate had been infused (7). This “twin” study showed that both the recruitment of fluid from the interstitial fluid space (*k*_21_) and the urine flow (*k*_10_) accelerated upon awakening from general anesthesia. Direct comparisons of the fluid distribution for 2.5% Glu+ and Ringer’s acetate when infused during the three studied phases are shown in Supplementary Figures S1, S2.

**FIGURE 4:**
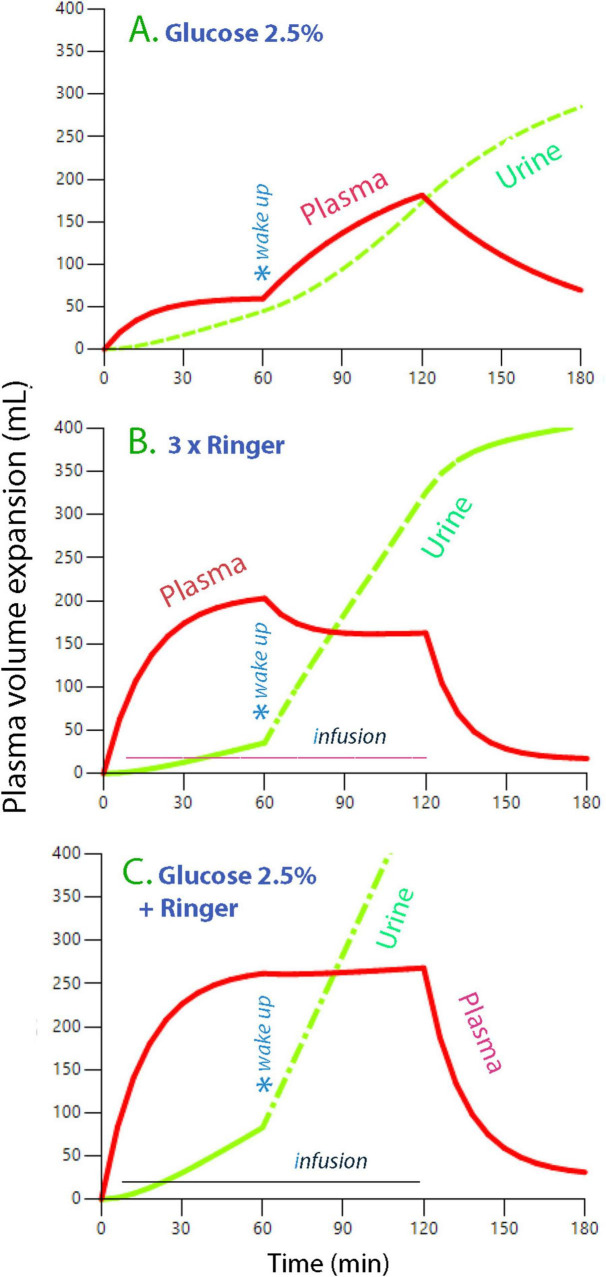
Simulations of 2.5% Glu+ and Ringer’s acetate. Plasma volume expansion and urine output when 2 L of fluid is infused at a constant rate over 2 h and wakeup from general anesthesia occurs at 1 h. One-fourth of the fluid volume consists of 2.5% Glu+ and three-quarters is Ringer’s acetate. **(A)** The contribution of 2.5% Glu+, **(B)** the contribution of Ringer’s, and **(C)** The combined effect of the two fluids. The maximum plasma glucose obtained with this fluid program was + 2.3 mmol/L.

Combinations of 2.5% Glu+ and Ringer kinetics suggested that an infusion matched to provide 25% of 2.5% Glu+ and 75% of Ringer’s would maintain a stable plasma volume both during and after surgery (Figure 4).

## 4 Discussion

### 4.1 Key findings

The present study adds to previous knowledge by describing and quantifying the glucose and volume kinetics occurring during the intraoperative and early postoperative periods. The alteration in the kinetics due to the changeover from the anesthetized to the postoperative state was analyzed in the same patients using covariates. The results also show how these two settings relate to the glucose and volume kinetics in awake volunteers not subjected to anesthesia and surgery.

The fluid volume kinetics differed markedly between the three studied settings (awake, anesthetized, and postoperative), while the glucose kinetics differed only between the awake and the other two settings. The primary hypothesis was then confirmed for the volume kinetics, but not for the glucose kinetics. The secondary hypothesis was confirmed for both the volume and the glucose kinetics.

If given in large amounts, the 2.5% Glu+ and Ringer combination apparently cause different types of edema as each fluid distributed differently. The glucose passes into the cells and adds water to the intracellular space either directly (by the osmotic force of the glucose) or after metabolism of the glucose molecules. By contrast, Ringer distributes only to the expandable parts of the extracellular fluid. Hence, 2.5% Glu+ causes both extra- and intracellular edema, whereas Ringer’s only expands the extracellular space.

### 4.2 Volume kinetics

Volume kinetics is the pharmacokinetics of the volume component of infusion fluids, but the calculations use plasma dilution instead of drug concentrations. The method identifies fictitious “walls” that delay the distribution of administered fluid. These walls represent functional barriers, but they may be interpreted in quasi-physiological terms as the crystalloid fluid volume that is not metabolized or bound to any tissue. Volume kinetics has been used to describe the kinetic characteristics of most infusion fluids and to provide answers to many physiological questions (16).

The present study identified a central volume of distribution for fluid (*V*_*c*_) of 3.12 L, which is close to the expected size of the plasma volume. The analysis further identified two extravascular fluid spaces: one situated close to the plasma and the other a more remote space (Figure 1). The latter space played a limited role in the present study and might represent, either in whole or in part, the intracellular space, as glucose brings along water when it enters the cells.

The covariate analysis identified the following three kinetic characteristics of 2.5% Glu+ during anesthesia that have previously been found for Ringer’s solution: (i) the rate constant for urine output (*k*_10_) increases with the arterial pressure (17), (ii) anesthesia accelerates the distribution of the infused fluid (*k*_12_) (6), and (iii) anesthesia retards the return flow to the plasma (*k*_21_) (18). The third effect agrees with experimental findings that both intravenous and volatile anesthetic agents inhibit lymphatic pumping (19).

The most important covariate effects observed during the postoperative period were the resolution of most of the anesthesia-induced inhibition of *k*_21_ and the addition of a strong inhibition of *k*_12_. Both of these covariate effects promoted plasma volume expansion (Figure 3C). The inhibitory effect of awakening on *k*_12_ can probably be explained by a decrease in capillary hydrostatic pressure due to restoration of the adrenergic tonus of the pre-capillary sphincters. Moreover, the capillary area for fluid exchange might decrease when the vasodilatation resolves. These three physiological changes occurred upon awakening, and all promote hypervolemia. Other factors that might have contributed to the hypervolemic effect include discontinuation of the pneumoperitoneum and removal of the patients from mechanical ventilation. The patients were also returned from the reverse Trendelenburg position to the flat recumbent position.

The simulations illustrated that the distribution of the fluid volume in 2.5% Glu+ differed in several ways from the plasma glucose scenario. In healthy volunteers, half of the infused volume remained in the body at a time when all glucose had been metabolized (cf. Figure 2C with Figure 3A). During anesthesia, the infused volume resided primarily in the extravascular space, from which it provided limited plasma volume support despite elevations in plasma glucose.

### 4.3 Plasma glucose

The difference in plasma glucose between volunteers and surgical patients is due to insulin resistance that develops in response to fasting and trauma. This hyperglycemic effect becomes more intense with extensive surgery (5, 20), although high-level epidural analgesia, which was not used here, might offer relief (21, 22). In a previous study, laparoscopic cholecystectomy was associated with a gradual 63% reduction in glucose clearance (20). In hip surgery patients, insulin sensitivity decreased by 45% from before to after hip replacement (5).

The maximum recommended infusion rate of 2.5% Glu+ should be determined by the induced rise in plasma glucose. In the present study, a bolus infusion of 500 mL of 2.5% Glu+ over 30 min was sufficient to cause a brief rise in plasma glucose to 10 mmol/L, with no difference between the surgery and the early postoperative periods. This conclusion is based on the similar strength of the covariance values that modified *k*_10_ during the intra- and postoperative periods compared to the awake setting. Starting at a steady state of 5 mmol/L, the plasma glucose level of 10 mmol/L would be reached by increasing the plasma glucose concentration by another 5 mmol/L (Figure 2C). This rise, if sustained, increases the risk of infection (23–25).

The central volume of distribution (*V*_*c*_) of glucose was 3 times larger than the *V*_*c*_ of the fluid (cf. Tables 2, 3). Administered glucose apparently distributes quickly across most of the extracellular space, while this process takes some time for the infused fluid volume (about 30 min). The reason probably involves a delay in the distribution of the fluid by the vascular wall.

### 4.4 The plasma glucose target

Ringer’s solution causes a minor dilution of plasma glucose (9), but the risk of hypoglycemia is linked to insufficient glycogenolysis or gluconeogenesis. Hence, a small risk of hypoglycemia arises during lengthy surgery in healthy adults. Patients with diabetes are at higher risk, as their metabolism might be hampered by the residual effects of antidiabetic medication (such as metformin). However, the optimal plasma glucose level during surgery in non-diabetic adults remains controversial.

Duncan et al. found that the incidence of complications after cardiac surgery was lowest when the plasma glucose varied between 7.8 and 9.4 mmol/L (26). Due to the risk of infection, the target in non-cardiac surgery is a plasma glucose of < 10 mmol/L, and no better outcome is achieved by adopting the tighter target of 5.5–7.7 mmol/L (27). Many surgical complications are stressful and may alone induce a rise in plasma glucose (25). Glucose levels of 12–15 mmol/L initiate osmotic diuresis, and even higher plasma glucose concentrations may impair cerebral outcomes after cardiac arrest (28, 29).

The possibility of alleviating insulin resistance by ingesting carbohydrates has been suggested (30), but our studies do not support this proposal (5, 31). Therefore, we must still surmise that surgery, even of a limited magnitude, reduces the capacity to eliminate glucose.

The present recommendation that an infusion of 2.5% Glu+ in adults should not exceed 500 mL over 30 min during the perioperative period is valid for laparoscopic cholecystectomy and probably also for surgeries with similar invasiveness. Patients undergoing more traumatic and major surgeries will have a poorer tolerance for glucose, whereas awake adults can metabolize substantially more glucose.

Due to the rise in plasma glucose, the administered fluid volume of 2.5% Glu+ is usually not large enough to fulfill the need for volume expansion during moderately large and major surgeries. We even identified a trend toward hypovolemia if an infusion of 2.5% Glu+ is given alone during surgery and is discontinued early. Any “free interval” between infusion and awakening appears to result in poorer central volume expansion. Therefore, the impact of waking up from general anesthesia on fluid distribution should be evaluated together with the influence of near-isotonic electrolyte solutions, such as Ringer’s and isotonic saline, on the same event (Figures 3E,F).

Combining the present kinetic data with those of a “twin” study using Ringer’s (7) suggested that providing a constant-rate infusion of both fluids during and after surgery is a good choice. The volume of the two fluids distributes similarly during general anesthesia, with both having a strong tendency to accumulate in the fast-exchange interstitial fluid space (Supplementary Figures S1, S2). However, they complement each other during the early postoperative phase because the effective restoration of *k*_10_ (urine output) with Ringer’s prevents the hypervolemic tendency with 2.5% Glu+, which is, in turn, due to low *k*_12_ and maintained low *k*_10_ (Figure 4). Conversely, *k*_21_ is restored by approximately the same magnitude using either fluid.

### 4.5 Fluid therapy after surgery

The Enhanced Recovery After Surgery (ERAS) program encourages the earliest possible return to oral intake of fluid (32), which can be 5–6 h after laparoscopic cholecystectomy. Whether intravenous fluid should be administered during that period remains controversial. A generally restrictive approach is recommended (33), and even more specifically after laparoscopic cholecystectomy (34), although a randomized trial by Vermeulen et al. reported that more complications occurred when fluid was restricted (35).

Urine flow is quite effective after laparoscopic cholecystectomy when Ringer’s is infused (7) and might even surpass that of a control infusion given before the surgery (36). In the present study, the urine flow was greater with 2.5% Glu+ than with Ringer’s during anesthesia but it increased markedly during the postoperative period for both fluids (Figure 4). Therefore, the use of restrictive fluid administration after this operation can be questioned.

### 4.6 Limitations

Patients undergoing laparoscopic cholecystectomy are placed in the reverse Trendelenburg position, with the head lifted slightly above the legs (usually at 20°). Part of the low *k*_21_ during anesthesia can probably be explained by a gravitational effect due to this body position (37).

The postoperative fluid kinetics were analyzed with good precision, although no fluid was infused during that period. Therefore, the simulations showing a strong plasma volume–expanding effect with 2.5% Glu+ after surgery assume that the fluid was also infused during the surgery. Whether the fluid has the same effect if given only during the postoperative period, as implicated in Figure 3C, is not known.

The present report uses data from three previous publications performed by the same research team using the same sampling protocol. The infusion rates used were higher than the recommendations that we give, but the glucosuria reported in the original studies was small and not sufficient to influence the kinetics (8–10).

The strengths of the present study include the ability of the kinetic analysis to recreate the measured data (Figure 1). Moreover, the postoperative kinetics were determined using the same patients as the anesthetized controls, although no fluid was infused during the postoperative period. We believe that the results are generalizable to all patients who undergo laparoscopic cholecystectomy.

The “twin” study on Ringer’s solution used acetate instead of lactate as a buffer. Acetate dominates in Scandinavia. It acts faster than lactate (38), requires less oxygen for its metabolism, and can be metabolized by all body cells, whereas lactate is only metabolized in the liver and kidneys. Acetate is more vasodilatory than lactate in large amounts, but the differences between acetate and lactate as buffers are minimal in clinical practice. They share the same volume kinetics (39).

## 5 Conclusion

Infusion of 2.5% Glu+ promotes hyperglycemia, which restricts the recommendable infusion rate during the perioperative period to 500 mL over 30 min. Kinetic analysis of the fluid volume showed that anesthesia inhibits the return flow of distributed fluid to the plasma, thereby promoting edema. This development was arrested by retardation of the fluid distribution during the postoperative period, which, in turn, increased the plasma volume expansion. Infusing Ringer’s solution along with 2.5% Glu+ would appear to offer a more stable volume distribution in patients waking up from anesthesia.

## Data Availability

The original contributions presented in the study are included in the article/Supplementary material, further inquiries can be directed to the corresponding author.
