# Supplementary material for: Influence of awakening from general anesthesia on the distribution of glucose 2.5% with balanced electrolytes in adults
Source: Front Med (Lausanne). 2025 Jun 4;12:1577418. doi: 10.3389/fmed.2025.1577418 (PMC12174063; doi:10.3389/fmed.2025.1577418)
Supplement: Supplementary file 1 [file Data_Sheet_1.docx]

**Supplementary file 1**

Influence of awakening from general anaesthesia on the distribution of

glucose 2.5% with balanced electrolytes in adults

***Kinetic model for fluid volume***

The Akaike criterion was -2628 (three-volume model), -2445 (two-volume) and -1964 (one-volume model). Hence, the three-volume kinetic model was chosen to analysis and simulation of fluid volume changes. This model is illustrated graphically in **Fig. 1A.** Fluid is infused into the plasma (*V*_c_) and distribution occurs to a fast-exchange interstitial space (*V*_t1_) from where it either returns to *V*_c_ or becomes further distributed to a more remote interstitial, slow-exchange fluid space (*V*_t2,_ the “third fluid space”).^9^ The elimination rate constant (*k*_10_) was calculated as the collected urine volume divided by the area under the curve of the volume expansion of *V*_c_.

This model is described by the following differential equations, using the symbolism was adopted from Gabrielsson J, Weiner D. *Pharmacokinetic & Pharmacodynamic Data Analysis: Concepts and Applications*. 4^th^ ed. Swedish Pharmaceutical Press 2006, pp. 80-82:

 d*v*_c_ /dt = *R*_o_ – *k*_12_ (*v*_c_ – *V*_c_) + *k*_21_ (*v*_t1_ – *V*_t1_) – *k*_10_ (*v*_c_ – *V*_c_)

d*v*_t1_ /dt = *k*_12_ (*v*_c_ – *V*_c_) – *k*_21_ (*v*_t1_ – *V*_t1_) – *k*_23_ (*v*_t1_ – *V*_t1_)

d*v*_t2_ /dt = *k*_23_ (*v*_t1_ – *V*_t1_) – *k*_32_ (*v*_t2_ – *V*_t2_)

dU /dt = *k*_10_ (*v*_c_ – *V*_c_)

where the three fluid compartments are *V*_c_, *V*_t1_, and *V*_t2_ at baseline and their expanded volumes are denoted by lower-case letters (*v*_c_, *v*_t1,_ and *v*_t2_). Hence, volume expansion of the central fluid space is given by (*v*_c_ – *V*_c_) and the flow of fluid from *V*_c_ to *V*_t1_ by *k*_12_ (*v*_c_ – *V*_c_). U is the collected urine volume. As a principle, the flow between compartments is obtained as the product of a rate constant signifying the flow and the volume expansion of the body fluid compartment from where the flow originates. Hence, the flow varies over time in proportion to the filling of that fluid space.

The Hb-derived fractional plasma dilution used to indicate the volume expansion of *V*_c_ resulting from the infusion. The plasma dilution was based on the relative reduction of the haemoglobin concentration (Hb/hb, "haemodilution") due to addition of cell-free fluid.

(*v*_c_ – *V*_c_) / *V*_c_ = [(Hb / hb) – 1)] / (1 – Hct)

where Hct is the baseline haematocrit, Hb is the blood haemoglobin concentration at baseline, while hgb refers to blood haemoglobin measured at a later time t. A minimal correction of the dilution was applied to account for surgical haemorrhage, if any, and the blood sampling (see *Anesth Analg* 2021; 133: 413–422).

In the analysis of plasma glucose, the two-compartment model was somewhat stronger than the one-compartment model (Akaike criterion 4270 *versus* 5252). However, the one-compartment model was chosen because *V*_c_ had a size of only 1.6 L and was not considered to adequately represent the plasma glucose concentration.

***Covariate analysis***

Kinetic differences between the awake and anesthetized state were examined and quantified by *covariates* *analysis.* Identification of appropriate covariates was guided by plots of random effects ("eta:s") (see Owen JS, Fiedler-Kelly J. *Introduction to population pharmacokinetic/pharmacodynamic analysis with nonlinear mixed effects models.* Hoboken, NJ; Wiley & Sons; 2014).

The *exponential covariate model* was used to analyse the influence of dichotomized variables on the parameters in the base model. For example, the rate parameter *k*_21_ has the group value of 0.0128 min^-1^ for the awake subjects. However, the value differs during the postoperative period, with a computer-generated covariate effect of -1.92. The equation for *k*_21_ for an individual (ind) then becomes:

*K*_21_ _ind_ = *k*_21_ _group_ [e ^awake=0, anaesthesia = -1.92^]

where e = 2.718. In an awake subject, *k*_21_ collapses to the group value of 0.0128 min^-1^ because (e^0 = 1). By contrast, *k*_21_ becomes 0.0128* e^-1.92^ ) = 0.00188, which is only 15% of the value obtained in the conscious subjects.

Continuous variables were evaluated according to the *power model*. For example, the mean arterial pressure averaged 86.6 mmHg, the group value for *k*_10_ was 0.0162 min^-1^, and the covariate effect was 8.44. The individual value of *k*_10_ for a subject with a MAP of 95 mmHg then becomes:

*k*_10_ _ind_ = 0.0162 [(95 / 86.6) ^8.44^ ] = 0.0354

The criterion for accepting a covariate was that its inclusion should reduce the -2 LL (log likelihood) for the model by > 3.8 points (*P*< 0.05) or > 6.6 points (*P*< 0.01). In addition, the 95% CI for the estimate of the covariate was not allowed to include 0.

***Kinetic model for glucose***

The kinetics of the infused glucose molecules was analysed using a one-compartment model in which the elimination rate is proportional to the plasma glucose concentration (C) at any time t:

C_t_ = C_o_ e ^-k10 t^

where C_o_ is the plasma glucose concentration extrapolated to zero time and *k*_10_ the elimination rate constant. The change in C at time t in response to an infusion given at the rate R_o_ is written as:

dC / dt = *R*_o_ – *k*_10_ C_t_

Analysis and simulations were performed with the same methodology and software as was used for the volume kinetic analysis.

***Ringer study***

Simulations based on the data from a ”twin study” where Ringer´s acetate had been infused were compared to the 2.5% glucose solution in **Fig. S1** and **S2** (overleaf). Just as for 2.5% glucose, covariate analysis showed that, when Ringer was used, *k*_21_ was significantly lower during the anesthesia compared to the fully awake state and the postoperative period. The urine flow was strongly depressed during anesthesia but resolved quickly on awakening and resumed the same value as in the fully awake state (BJA Open 2022; 2: 100013).


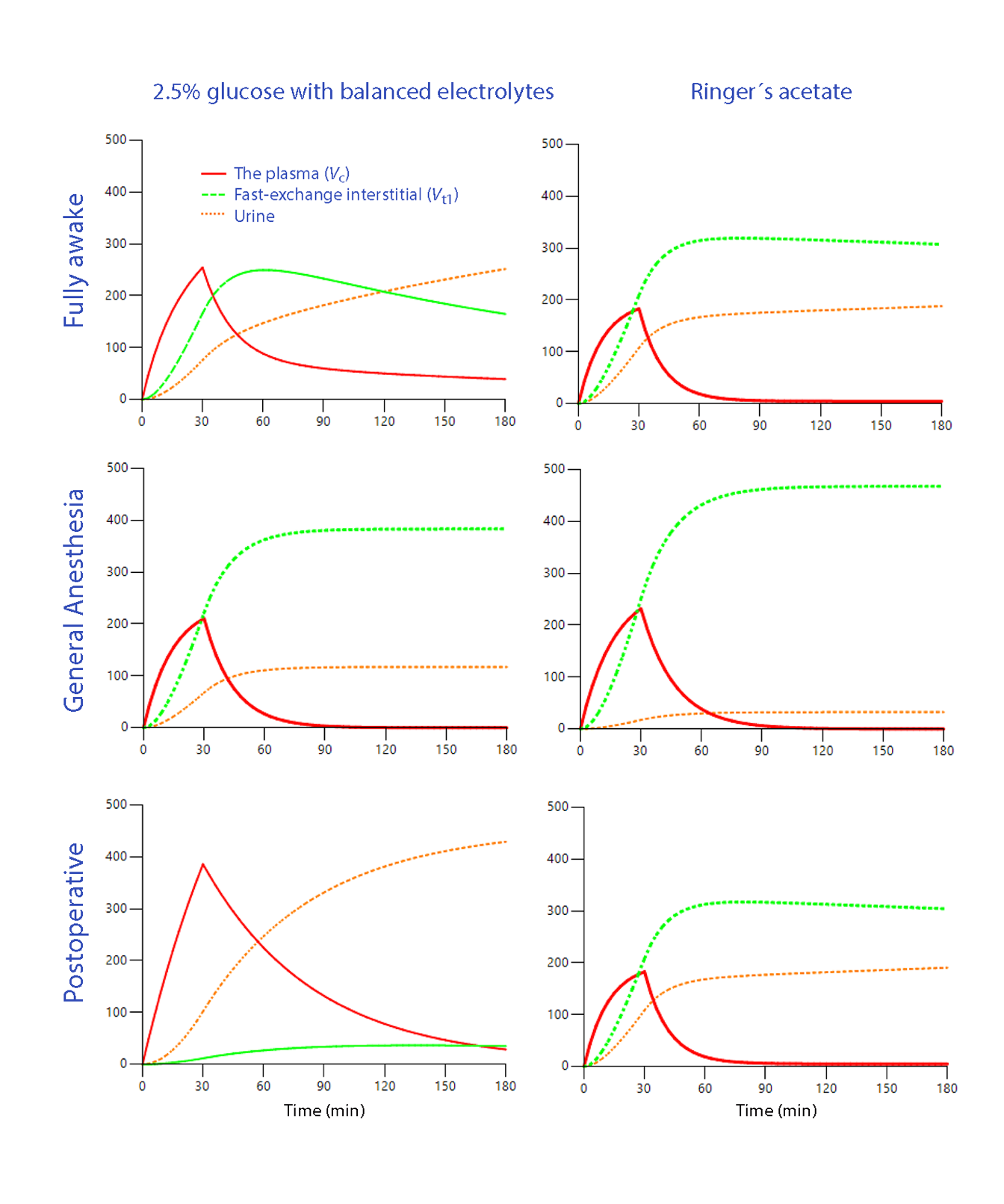


**Fig. S1.**

**Simulations of 2.5% glucose with balanced electrolytes *versus* Ringer´s acetate.**

Plasma volume expansion (*V*_c_), volume expansion of the fast-exchange interstitial fluid space (*V*_t1_), and urine output during 3 h when 500 mL of 2.5% glucose with balanced electrolytes (left columns) and Ringer´s acetate (right columns) is infused over 30 min in the fully awake state (top row), during general anesthesia (middle row) and during the first 2 h postoperatively (bottom row). Kinetic data for the glucose infusions were derived from Table 2 of the present study and the kinetic parameters for Ringer´s acetate from Ref. 7

**
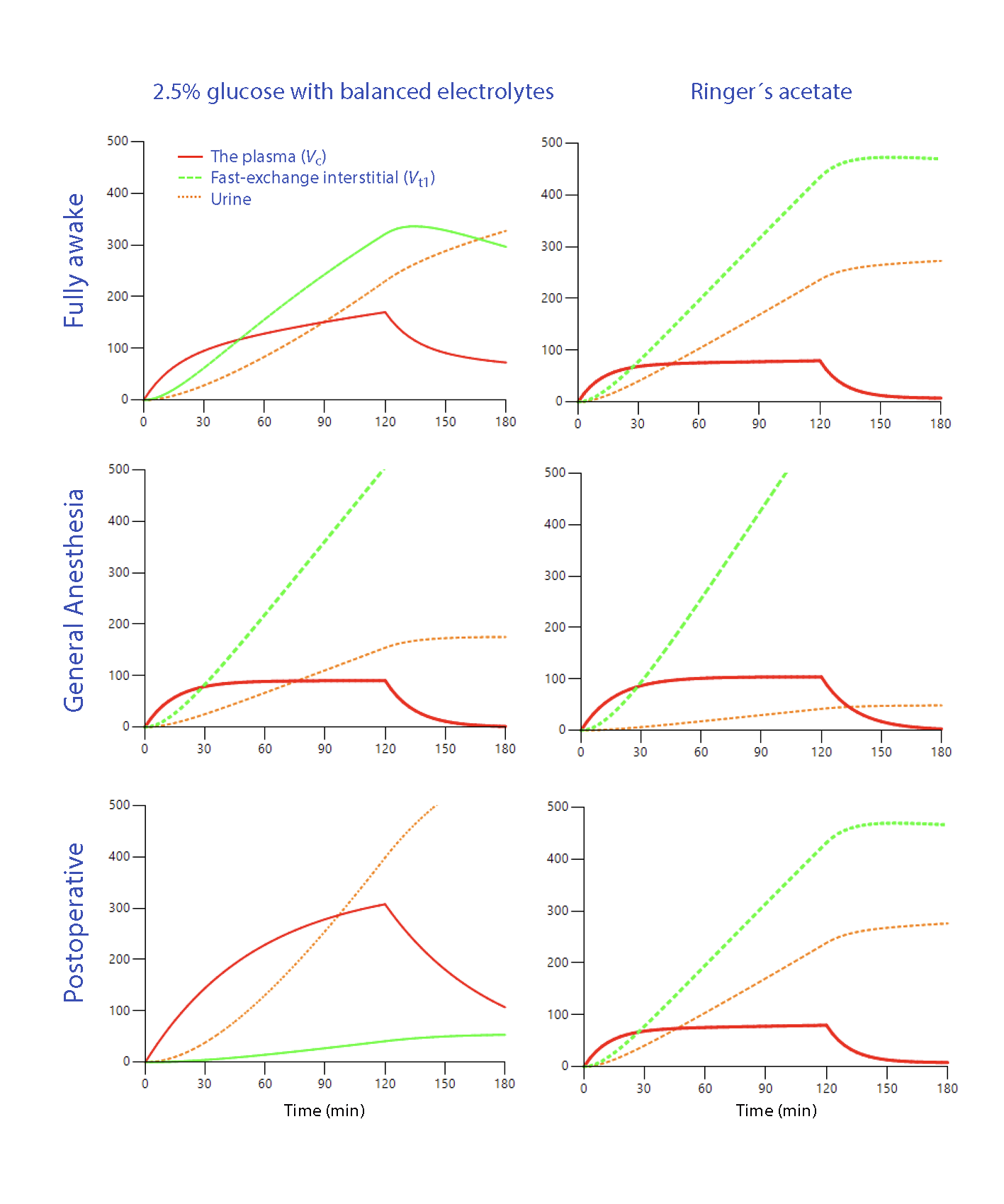
**

**Fig. S2.**

**Same plot as Fig. S1 but 750 mL of fluid is infused over 2 hours**. Kinetic data for the glucose infusions were derived from Table 2 of the present study and the kinetic parameters for Ringer´s acetate from Ref. 7
